# The Impact of COVID-19 Restrictions and Changes to Takeaway Regulations in England on Consumers’ Intake and Methods of Accessing Out-of-Home Foods: A Longitudinal, Mixed-Methods Study

**DOI:** 10.3390/nu15163636

**Published:** 2023-08-18

**Authors:** Mackenzie Fong, Steph Scott, Viviana Albani, Heather Brown

**Affiliations:** 1 National Institute for Health and Care Research Applied Research Collaboration (North East and North Cumbria), Newcastle-upon-Tyne NE3 3XT, UK; 2 Population Health Sciences Institute, Faculty of Medical Sciences, Newcastle University, Newcastle-upon-Tyne NE1 7RU, UK; 3 Division Health Research, Faculty of Health and Medicine, Lancaster University, Lancaster LA1 4AT, UK

**Keywords:** food delivery services, takeaway, fast food, food environment, town planning, obesity

## Abstract

Background: COVID-19 restrictions significantly impacted the operations of fast food and full-service retailers. Full-service retailers were permitted to operate as takeaway outlets without needing to seek formal changes in planning permissions. We conducted a study to determine consumers’ intake and modes of accessing foods from fast food and full-service retailers during various COVID-19 restrictions and changes to takeaway/delivery regulations, as well as their experiences. Methods: We conducted a longitudinal, mixed-methods study comprising three surveys, which examined the intake frequency and modes of accessing retailers, and two rounds of qualitative focus groups, which explored their related experiences. The data were collected at three timepoints (T) from May 2021–March 2022. The participants were adults living in Northern England (*n* = 701 at T1); a sub-sample participated in the focus groups (*n* = 22). The intake data were presented descriptively; an ordered logit regression explored the factors associated with the intake frequency. The focus group data were analysed using a framework analysis. Results: The mean weekly intake frequency from fast food retailers at T1, T2, and T3 was 0.96 (SD 1.05), 1.08 (SD 1.16), and 1.06 times (SD 1.12), respectively. For full-service retailers, this was 0.36 (SD 0.69), 0.75 (1.06), and 0.71 (SD 0.99) times, respectively. Food access issues (OR (SE): T1 = 1.65 (0.40), T2 = 2.60 (0.66), T = 2.1 (0.62)) and obesity (T1 = 1.61 (0.31), T2 = 2.21 (0.46), T3 = 1.85 (0.42)) were positively associated with intake from fast food, but not full-service retailers. Delivery services were commonly used to access fast food (30–34% participants), but not full-service retailers (6–10% participants). As COVID-19 restrictions eased, participants were eager to socialise on-premises at full-service retailers. Conclusions: Takeaway/delivery services were seldom used to access full-service retailers, but the use of delivery services to access fast food was high. Policymakers must recognise delivery services as a growing part of the food environment, and the challenges they pose to planning policies for obesity prevention.

## 1. Introduction

On 23 March 2020, England entered its first national ‘lockdown’ to attenuate the spread of COVID-19 [1]. Legally enforced ‘stay-at-home’ orders meant that people could only leave their homes for essential activities, and non-essential businesses were not permitted to conduct on-premises trade [1]. Since then, COVID-19 restrictions in England have fluctuated [2] in response to factors, including case numbers, hospitalisation rates, and disease severity, among others. These restrictions had a particularly large impact on the out-of-home (OOH) food sector and affected both dine-in (food eaten on-premises) and takeaway/delivery (food eaten off-premises) operations. Regarding dine-in, between intermittent national and local lockdowns during which they were prohibited, the phased reopening of dine-in services was accompanied by varying public health measures [2]. For instance, retailers were only able to accommodate patrons in outdoor spaces, and patrons were required to wear face coverings while not seated, adhere to social distancing, restrict social contact between households (‘Rule of Six’), and ‘check-in’ to venues on the NHS COVID-19 app. In February 2022, the U.K. Government moved to the ‘Living with COVID-19’ plan where all legal limits on business operations and social contact were abandoned [3]. 

With regards to takeaway and delivery operations, in March 2020 the Ministry of Housing, Communities, and Local Government announced temporary changes to town planning regulations that allowed retailers, cafes, pubs, and bars (A3 and A4 Use Class retailers [4], herein collectively referred to as full-service retailers) to use ‘enhanced’ takeaway and delivery services without seeking formal changes in planning permissions [5], meaning that most food sold could be ‘taken away’ to be eaten off-premises. Prior to the pandemic, full-service retailers only used takeaways/delivery services in a supplementary capacity. The ‘enhanced’ use of these services was reserved for hot food takeaways (A5 Use Class 1; herein referred to as fast food retailers) only. While these changes to planning regulations were introduced to support ‘stay-at-home’ orders and preserve the economy and employment, town planning and public health stakeholders expressed concern that they were incompatible with public health agendas and could have implications for population-level weight gain by facilitating access to OOH foods [6]. These concerns were plausible given that greater access to OOH foods, characteristically very high in energy [7,8], has been linked to greater consumption and higher BMIs in some studies [9,10]. For instance, a population-based, cross-sectional study found that objectively measured exposure to home, workplace, and commuting route food environments were associated with a marginally higher consumption of takeaway food, greater body mass index, and greater odds of obesity, with evidence of a dose-response effect [10]. Given the association of obesity and socioeconomic deprivation [11], and that the density of fast food outlets is greater in areas with higher deprivation levels in the U.K., such as northern England [12], negative health repercussions of policies affecting access to OOH foods could be more pronounced in areas with greater deprivation.

Understanding how changes to takeaway regulations and various COVID-19 restrictions over the course of the pandemic in the U.K. may have impacted how often consumers ate foods from different OOH retailers, how they accessed them, and their associations with sociodemographic factors would help inform policies on the OOH food sector and future crisis planning. To our knowledge, no previous U.K.-based studies explored this topic. While survey studies have quantified OOH intake during the pandemic, they were not conducted over sufficient timeframes to explore the impacts of the various restrictions [13,14,15], nor did they differentiate intake from the different types of OOH retailers [13,16,17]. We were not aware of any studies that investigated how consumers accessed OOH retailers across the course of the pandemic, nor consumers’ use of the newly introduced ‘enhanced’ takeaway services for full-service retailers. One U.K.-based study aimed to explore the associations between changes in BMI and different food outlets/methods of delivery in a small sample of U.K. adults (*n* = 60) [18]. However, the type of food outlets were reported as fast food restaurants, full-service restaurants, delivery, or takeaways. These options conflated the retailer type with the method of access since both types of retailers could operate as ‘takeaways’ during the pandemic, limiting confidence in the findings. Additionally, notwithstanding one mixed-methods study exploring consumers’ experiences of home cooking and eating out in the U.K. during the first months of lockdown [19], we were not aware of any other qualitative work exploring consumers’ perceptions and experiences of using the OOH food sector and new takeaway services during various COVID-19 restrictions across the course of the pandemic. 

Therefore, we co-conceived a study with public health stakeholders in Northern England that aimed to investigate the use of OOH food retailers by consumers in Northern England during various COVID-19 restrictions and changes to takeaway regulations from May 2021 to March 2022. The specific objectives were as follows.

1. To determine the frequency of intake from fast food and full-service retailers.
2. To determine the association of intake from fast food and full-service retailers with sociodemographic characteristics to understand the variations across the socioeconomic spectrum.
3. To determine how food from fast food and full-service retailers was accessed and why.
4. To explore consumers’ experiences and perspectives of using OOH food retailers.

## 2. Materials and Methods

### 2.1. Research Paradigm and Study Design

**Figure 1:**
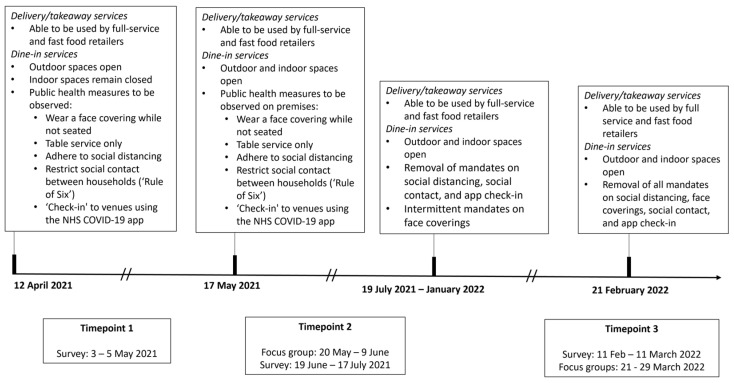
Timeline of the study data collection, COVID-19 restrictions, and adaptations affecting OOH fast food and full-service retailers in England from April 2021–March 2022.

Since our research objectives lent themselves to both positivist (objective, quantitative) and interpretivist (subjective, qualitative) paradigms, we selected a pragmatic paradigm. Pragmatism acknowledges that research inquiries can be answered by drawing from different research paradigms [20] and through the use of quantitative, qualitative, and mixed research [20]. We conducted a longitudinal, mixed-methods study comprising three online quantitative surveys and two rounds of qualitative focus groups conducted with adults residing in Northern England (Figure 1). A convergent parallel mixed-methods design was used, in which qualitative and quantitative data were collected in parallel, analysed separately, and then merged [21]. This was performed at a slight lag as the initial survey was used to identify the potential focus group participants.

### 2.2. Study Context

The study period was from May 2021 to March 2022, during which time COVID-19 restrictions affecting the OOH food sector in England fluctuated. At data collection timepoint 1 (T1) from 3–5 May 2021, OOH retailers were allowed to provide dine-in services in outdoor spaces only [2]. Patrons were required to wear face coverings while not seated at a table, use table service only, adhere to social distancing, restrict social contact between households (‘Rule of Six’), and ‘check-in’ to venues using the NHS COVID-19 app. At timepoint 2 (T2) from 20 May–17 July 2021, indoor spaces were reopened, but public health measures were still enforced [2]. At timepoint 3 (T3) from 11 February–29 March 2022, the U.K. Government moved to the Living with COVID-19 plan and all legal limits on OOH business operations and social contact were abandoned [3]. With regards to takeaway/delivery operations, both fast food and full-service retailers were able to use these services throughout the study period, i.e., both were able to sell most food as ‘takeaway’ to be eaten off-premises. 

### 2.3. Surveys

#### 2.3.1. Procedure for Survey

The survey was piloted before the formal data collection and amended according to the feedback. The participants were invited to complete the baseline survey at T1. Those who completed it were invited to complete follow-up surveys at T2 and T3. Relative to the survey administered at T1, the surveys administered at T2 and T3 were live for longer to maximise the participant response rate. Each participant was paid GBP 2.25 for completing the survey at T1 (the equivalent of GBP 9.00/h for a 15-min survey) and GBP 1.55 for completing surveys at T2 and T3 (the equivalent of GBP 9.30/h for a 10-min survey). Payment was submitted directly to the participants via Prolific [22], an online platform that connects researchers with participants.

#### 2.3.2. Participant Recruitment and Sampling for Survey

All the study participants were recruited via Prolific [22] and all the participants provided their consent before commencing each survey. The participants were ≥18 years and lived in Northern England. The recruitment was stratified in Prolific by age (18–24 years, 25–49 years, 50 years and over), sex (male/female), and annual household income (<£30,000 disposable household income, ≥£30,000 disposable household income informed by income by region) [23]. Since the analyses were exploratory, we did not conduct a formal power analysis, and we aimed for a sample size of 700 participants. A retrospective sample size calculation was conducted and showed that a sample of *n* = 700 would be sufficient to achieve a 5% margin of error with a 99% confidence interval. Additionally, as a rule of thumb for an ordered logit which we employed, a sample of *n* = 500 was the minimum size required to estimate the parameters [24]. Therefore, our sample was adequate for estimating the associations we investigated [24]. 

#### 2.3.3. Measures

An overview of the data collected at each timepoint is shown in Table 1. The participants’ age, sex, and annual household income was provided via Prolific. The study survey administered at T1 (Supplementary File S1) collected other demographic measures and habitual health behaviours, including self-reported height and weight, annual household income (the mean and median household incomes in 2019–2020 for North East England were GBP 23,800 and GBP 30,000, respectively [23]), employment status, ethnicity, education (A-levels; a U.K. high school leaving qualification), and experience of issues accessing foods due to financial constraints using the three-item U.S. Department of Agriculture household food insecurity screener [25].

The participants reported their usual fruit and vegetable intake via relevant items in a short-form dietary questionnaire [26], usual alcohol intake via the Alcohol Use Disorders Identification Test Consumption (AUDIT-C) [27], and usual physical activity via the International Physical Activity Questionnaire (IPAQ) short [28]. All these are validated instruments and have been widely used in health research to measure health behaviours. The participants then reported the frequency with which they consumed hot foods from OOH food retailers in the previous 7 days and how these foods were accessed (delivery, dine-in, takeaway). Since we wanted to explore the use of newly introduced ‘enhanced’ takeaway and delivery services by full-service retailers, OOH food retailers were dichotomised into fast food retailers (historically able to use ‘enhanced’ takeaway and delivery services) and full-service retailers (able to use ‘enhanced’ takeaway and delivery services since March 2020). Written descriptions and images of these two groups of retailers were provided to help the participants differentiate them. The instructions were explicit that we were interested in the intake of hot foods only, and not cold items such as crisps or confectionary. In the follow-up surveys at T2 and T3, the participants reported their use of fast food and full-service retailers, methods of accessing these retailers, alcohol use, and physical activity over the previous 7 days. All the surveys included attention check questions to identify any participants responding randomly.

#### 2.3.4. Data Analysis for Survey

We present a descriptive analysis in which we estimated the means for all the variables. For the continuous variables only (age, BMI, and MET hours per week of physical activity), the standard deviations were estimated. The continuous variables were interpreted as natural units whereas binary variables could be interpreted as percentages. Next, we employed an ordered logit regression to explore the factors associated with an increased frequency of consuming food from fast food and full-service retailers over the study period. This showed the association between the different levels of consumption of food from fast food and full-service retailers and the individual characteristics over the study period (the variables outlined in the descriptive statistics).

### 2.4. Focus Groups

#### 2.4.1. Procedure for Focus Groups

All the focus groups were conducted via a video conferencing platform (Microsoft Teams app version: 1449/1.0.94.2021121302) and were audio and video recorded. The groups were facilitated by MF and SS; both female research fellows educated to a PhD level with moderate to extensive experience in conducting qualitative research. MF had a dietetics background and an interest in obesity management/prevention. SS had a social science background with an interest in health inequalities. The participants were not known to the researchers before the study and were not explicitly informed of the researchers’ academic backgrounds or interests. Throughout the research process, the researchers reflected on their own experiences, interests, and views and how these might have influenced the conduct of the study, especially the analysis and interpretation of the focus group data. To ensure that online discussion was manageable, the focus groups were limited to a maximum of six participants. The participants could join using audio only or by using both video and audio. All the focus groups were steered by a topic guide (Supplementary File S2), which was initially devised in consultation with regional stakeholders and guided by both the existing literature and survey questions. This topic guide was iterative, allowing for space to continually re-evaluate the emergent findings and perspectives. All the participants were provided with a study information sheet and submitted written, informed consent to partake in the study before each focus group commenced. 

#### 2.4.2. Participant Recruitment and Sampling for Focus Groups

The participants indicated their interest in participating in a focus group at the end of the survey completed at T1. The focus group participants were identified using maximum variation purposive sampling based on their age, gender, and annual household income. All the focus group participants who took part at T3 had previously taken part at T2, resulting in a repeat focus groups. The focus group participants received a payment of GBP 15 per focus group, which was again submitted directly to their Prolific account. At both time points, the focus groups were conducted until data saturation was reached, whereby the existing themes were consistently repeated, and no new themes were drawn from the data [29].

#### 2.4.3. Data Analysis for Focus Groups

The focus group recordings were transcribed verbatim and anonymised. The transcripts were analysed using the five iterative stages of a framework analysis outlined by Ritchie and Spencer [30]: 1. familiarisation, 2. identifying a thematic framework, 3. indexing, 4. charting, and 5. mapping and interpretation. The codes were generated inductively and deductively as informed by the focus group topic guide. The transcripts were coded independently by MF and SS, firstly line-by-line and then systematically indexed into data tables to generate candidate themes. The candidate themes were discussed and challenged at subsequent project meetings, and were compared to identify patterns, similarities, and differences in the data in order to generate analytical themes and a consistent interpretation of the dataset as a whole. Since the data were longitudinal, we used a recurrent cross-sectional analysis, which allowed us to explore the evolution of group-level themes over time, rather than the trajectories of the individual participants [31]. 

### 2.5. Data Integration 

We used a side-by-side comparison approach to integrate the datasets of the convergent parallel design, as outlined by Creswell and Clark [21]. First, each dataset was analysed separately. Then, we compared our analysis of the survey data with the analysis of the focus groups, identifying the common concepts and convergence or divergence in findings. We grouped our findings under subheadings that were broadly based on our research objectives. Depending on the nature of the objective and in line with the pragmatic research paradigm, these contained quantitative survey data, qualitative focus group data, or both to elaborate on or corroborate the findings from both data sources. Further data integration is provided in the Discussion section to offer overarching take-home messages and/or formulate recommendations for policy and practice. To help illustrate the findings, we included graphs and quotations to provide rich description and faithful accounts of the views and experiences of the participants in this study.

## 3. Results

### 3.1. Participants

#### 3.1.1. Survey

A total of 874 participants were recruited at T1. Of these, 173 participants did not submit a complete survey, resulting in a sample of 701 participants at T1. The sample characteristics are reported in Table 2. The proportion of men and women (49.9%) was roughly equal. Most participants were white (90.6%), and approx. one-third (35.8%) had a basic level of education (high school or lower). The proportion of participants who had difficulty accessing food for financial reasons ranged from 8.6–14.7%, depending on the definition used (Table 2 footnote). At the baseline, 142 participants (20.3%) reported not drinking alcohol at all. Of those that reported drinking, 278 (49.7%) participants were classified as low-risk drinkers, 267 (47.8%) as increasing and higher risk drinkers, and 12 (2.5%) as having a possible dependence. Of the 701 participants in the initial sample, 615 (87.7%) and 490 (69.9%) participants completed the survey at T2 and T3, respectively. At T2 and T3, the participant composition remained similar to T1. However, fewer women and younger people responded in the later waves.

#### 3.1.2. Focus Groups

At T2, six focus groups were conducted with 22 participants consisting of two to six participants per group. The sample consisted of 10 male and 12 females with a mean age of 40 years (range = 21–65 years). Ten participants (45%) had an annual household income below the median annual earnings for full-time employees in Northern England [32] (Table 3). One participant was shielding since March 2020. Each focus group discussion lasted between 49 and 62 min. At T3, three focus groups were conducted with 12 participants from the original sample. The sample consisted of equal numbers of male and female participants with a mean age of 45 years (range = 24–62 years). Three participants (25%) had an annual household income below the median annual earnings for full-time employees in Northern England [32]. The focus groups at T3 lasted between 32 to 53 min.

### 3.2. Intake Frequency of OOH Foods

**Figure 2:**
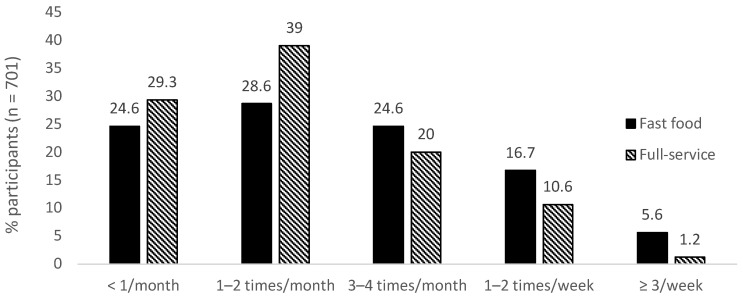
Intake frequency from fast food and full-service retailers before the pandemic.

**Figure 3:**
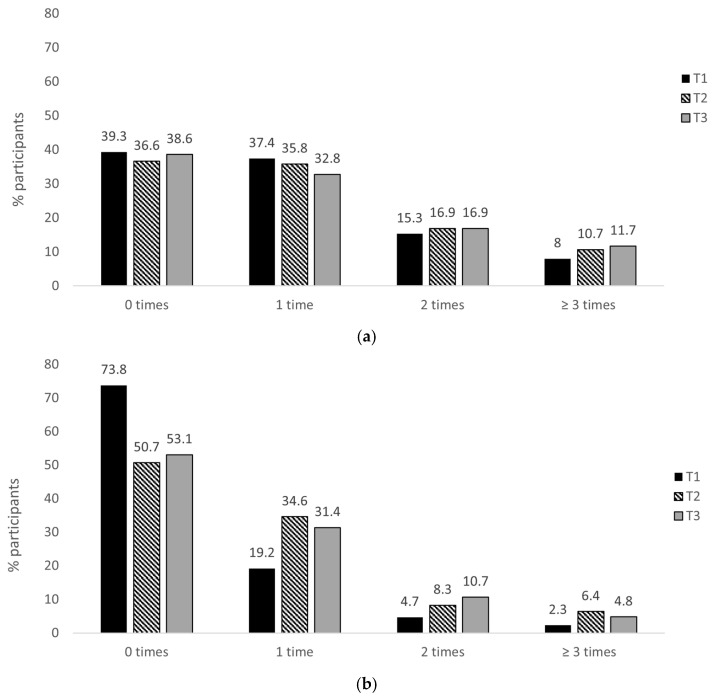
Weekly intake frequency across the time points of foods from (**a**) fast food retailers and (**b**) full-service retailers. Timepoint 1 (T1) from 3–5 May 2021: dine-in services in outdoor spaces only; public health measures enforced on-premises. Timepoint 2 (T2) from 20 May–17 July 2021: indoor spaces reopened; public health measures enforced on-premises. Timepoint 3 (T3) from 11 February–29 March 2022: all legal limits on OOH retailers abandoned. Takeaway/delivery services were available both for fast food and full-service retailers at all time points.

The greatest proportion of participants reported that their intake from both fast food (39.0%) and full-service (28.6%) retailers before the pandemic was one to two times per month (Figure 2). Overall, the pre-pandemic intake from fast food retailers was more frequent than the intake from full-service retailers (Figure 2). Similarly, at all three survey time points, the intake from fast food retailers was greater than that from full-service retailers (Figure 3a,b). The mean weekly intake frequency from fast food retailers at T1, T2, and T3 was 0.96 (SD 1.05), 1.08 (SD 1.16), and 1.06 times (SD 1.12), respectively. For full-service retailers, the mean weekly intake frequency was 0.36 (SD 0.69), 0.75 (1.06), and 0.71 (SD 0.99) times, respectively. The frequency distribution of the intake from both retailer types at each timepoint is presented in Figure 3a,b.

### 3.3. Intake of OOH Foods across Sociodemographic Groups

The survey data showed that several participant characteristics were consistently associated with the intake frequency from different retailers across all three timepoints (Table 4 and Table 5). The participants aged 18–24 years old used both fast food and full-service retailers significantly more than the participants in other age groups at all three timepoints. Likewise, those who experienced food access issues ate from fast food retailers 1.65 (SE 0.40), 2.60 (SE 0.66), and 2.1 (SE 0.62) times more than those without food access issues at T1, T2, and T3, respectively. The participants with obesity used fast food retailers 1.61 (SE 0.31) (T1), 2.21 (0.46) (T2), and 1.85 (0.42) (T3) times more than the participants with a healthy BMI. Neither food access issues nor obesity status were associated with use of full-service retailers. At T1, carers used fast food retailers 3.5 (SE 1.3) times more than non-carers. The reason for this was partially explored in the focus groups discussions. While some participants did not identify as ‘carers’ per se, several female participants with children mentioned that competing domestic and childcare duties took time and cognitive resource away from meal preparation, leading them to opt for fast food as a quick and easy alternative.

*“I’m so busy doing everything else, it tends to be now we will just go to McDonald’s on the way home and and then just grab something quick and easy in our house usually means something that’s like full of calories and fat just because it’s, it’s just easy to grab”*. (Female; 40–49 years; Focus Group at T3).

*“Yeah, so tired on a Friday to think about looking nice for a meal or booking something. You’re just like no it’s just that easy… you don’t even have to get out of your car, just drive by you’ve got your meal in 10 min”*. (Female; 40–49 years; Focus Group at T3).

At T2, the participants with the greatest annual household income used full-service retailers 2.4 (SE 0.73) times more than those with the lowest household income, an observation that approached significance at T3. These findings were corroborated by the focus groups discussions where several participants noted that prices at full-service retailers were higher than before the pandemic. Conversations surrounding cost were more central during the second round of focus groups, and many participants indicated that it played a significant factor in deciding whether and where to eat OOH. Some participants reported that they would prefer to use fast food retailers to limit expenses, or that using a full-service retailer would be saved for special occasions only.

*“Yeah, I think that the what’s more noticeable for us is how much more expensive things were than before COVID. So actually, the price plays more into our decision about whether we go out or have takeaway than the COVID restrictions and and sort of like living with COVID does it’s more about the price now… I can’t justify that on a very small household budget, so we will probably get more takeaways now as a treat.”*. (Female; 40–49 years; Focus Group at T3).

### 3.4. Methods of Accessing OOH Foods

**Figure 4:**
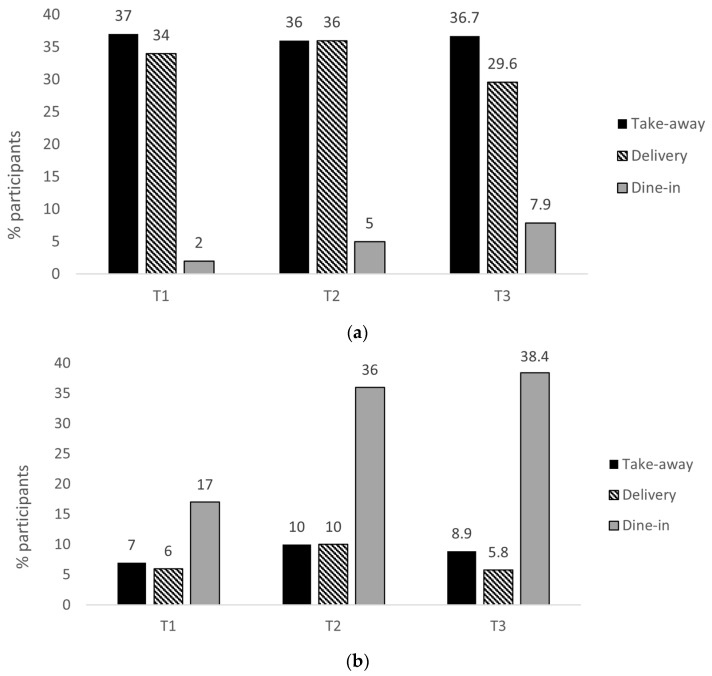
Methods of accessing food across the time points from (**a**) fast food retailers and (**b**) full-service retailers. Timepoint 1 (T1) from 3–5 May 2021: dine-in services in outdoor spaces only; public health measures enforced on-premises. Timepoint 2 (T2) from 20 May–17 July 2021: indoor spaces reopened; public health measures enforced on-premises. Timepoint 3 (T3) from 11 February–29 March 2022: all legal limits on OOH retailers abandoned. Takeaway/delivery services were available both for fast food and full-service retailers at all time points.

The survey data showed that takeaway and delivery services were the predominant method used to access fast food across all three timepoints (Figure 4a). In the focus group discussions at T2, some participants reported they preferred to have food delivered as this was perceived safer in terms of the COVID-19 infection risk, while others preferred to collect food from fast food retailers to ‘get out of the house’ and alleviate boredom during lockdown.

*“I mean it’s just a little bit like obviously, living in a house where I live with a friend, it’s just nice to get out of the house and just be together for a little bit. I would say it is just getting out of the house with us being in the house most of the time”*. (Male; 20–29 years; Focus Group at T2).

When discussing delivery services, many focus group participants felt that the use and presence of digital food delivery platforms (DFDPs), e.g., Uber Eats, Deliveroo, and delivery drivers had increased since the pandemic. While some enjoyed the increased variety and convenience these platforms afforded, others were concerned about how this would affect smaller, independent businesses, and remarked that they promoted overspending.

*“I ordered a McDonalds breakfast the other day because I had no coffee and I wanted a coffee [laughs] honestly, I thought I will get a McDonalds breakfast because you can get them delivered now with Just Eat or whoever, Deliveroo. I got two breakfasts and it came to like £12.00 and I thought, ‘What am I doing?’ I only wanted a coffee….And £12.00 so, I was thinking, how dan-gerous is it now though? Just to be able to just, I know you can always get takeaway food but there is so much more option now like McDonalds, I don’t know if that was during lockdown or whatever, but it seems like every food outlet now will have Deliveroo or Just Eat or whatever… people are going to want to go to the big boys rather than the little fellas mostly and it’s going to just take money out of their pocket but yeah, I think it’s quite dangerous the amount of availability that there is now.”*. (Male; 40–49 years; Focus Group at T3).

Some participants were enticed to use a DFOP for the first time during the study period due to introductory promotional offers.

*“I used to use Deliveroo sometimes. Uhm, I used it a couple of times because I think we got a leaflet through the door where you got 50% off or something. Umm which was really good. And Uber eats as well.”*. (Female; 50–59 years; Focus Group at T3).

The survey data showed that 54% of participants used new ‘enhanced’ takeaway/delivery services at least once to access full-service retailers since changes to the planning regulations were made in March 2020. However, the use of these services was relatively low across all three timepoints (6–10%) (Figure 4b). The reasons for this were discussed in the focus groups. Mainly, many participants stated they wanted a ‘traditional’ takeaway when having food to eat at home. Several participants also remarked that the quality of delivered food was generally perceived as poorer than that consumed when dining in (e.g., soggy, cold), and in some cases, this deterred participants from using delivery services again. While the participants spoke of the positive aspects of the introduction of takeaway/delivery services for full-service retailers, e.g., discovering new retailers and supporting businesses, there was consensus that these services were no longer needed when dine-in operations resumed (except for a slightly divergent view from the participant who was shielding and who still valued being able to have food from full-service retailers delivered to their home). The participants wanted to eat on-premises and recapture the social aspect of eating out that was greatly missed during the lockdowns. Full-service retailers were inextricably linked to socialising with friends and family, usually to mark special occasions, e.g., birthdays, anniversaries, or end of exams.

*“I think it’s about family and friendships and eating out is usually around an occasion to be fair…We like to get dressed up and just make a real night of it…it’s about, for us it’s about massively the social side of it.”* (Female; 20–29 years; Focus Group at T2).

*“…eating out was always about being with other people, like not just going out to get some food. It’s more about the experience of being around the people you like spending time with...”* (Male; 20–29 years; Focus Group at T2).

When reflecting on the lockdowns and being unable to dine-in at full-service retailers, the participants primarily spoke about missing the social aspect of eating out and getting dressed up, rather than the food. During the second round of focus groups (T3), being able to go to full-service retailers without restrictions allowed the participants to reconnect with family/friends from whom they felt distanced during lockdown. These focus group findings corroborated the survey data showing that dine-in was the most common method for accessing full-service retailers (Figure 4b) and that the use of dine-in services increased markedly from 17% at T1 to 36% at T2 as restrictions eased and was sustained at T3 (38.4%). In contrast, fast food retailers were not discussed in the context of socialising and were primarily used as a means to obtain food conveniently and quickly and reduce ‘foodwork’, i.e., food preparation and washing up.

*“When you go into a retailer, you’re fulfilling a social need. When you have a takeaway, you’re having food”*. (Male; 20–29 years; Focus Group at T2).

### 3.5. Experiences of Dining in at Full Service Retailers

The analysis of the focus group data yielded two sub-themes related to the experience of dining in at full-service retailers: the perceptions of COVID-19 risk, and the enjoyability and atmosphere. Regarding the first theme, most of the participants reported that they felt some concern about COVID-19 risk while dining in at full-service retailers during the first round of focus groups. This concern was two-fold and related to the participants’ own vulnerability to contracting COVID-19, but also the risk of infecting others, especially older and more vulnerable people. The perception of COVID-19 risk was contingent upon macro-level factors, such as high case numbers, pervasive coverage in the media, population vaccination rates, and more proximal factors pertaining to individual venues such as crowdedness, patrons not observing social distancing, and unsatisfactory hygiene practices.

*“Yeah, even sitting outside, I’m just not comfortable because you don’t know people’s hygiene. You just don’t know what the cleaning regime is. You don’t know if that person sitting next to you has got COVID19 and yeah, had one vaccine, but you don’t know. You mightn’t have antibodies. You don’t know”*. (Female; 50–59 years; Focus Group at T2).

While some measures such as distanced seating and outdoor seating allayed anxiety for some participants, others remained concerned and preferred to wait until they perceived the COVID-19 infection risk to be lower before dining on-premises. 

At the time of the second focus groups (T3), virtually all the participants were unconcerned about COVID-19 risk when dining on-premises and this was attributed to greater population vaccination rates, having personally contracted COVID-19 previously, perceived conferred immunity, perceived mildness of the Omicron variant (the dominant variant at the time), and lower media coverage of COVID-19 despite that COVID-19 infection rates were still considered high by the participants. The participants considered their experience of dining in to be ‘back to normal’.

*“I am very much the same as [name] so, I have had all three jabs, I have also had Covid twice so, I now feel like I am pretty much Superman [laughs] so, it’s fine. Like, I have had it twice, it has not really bothered me either time so, I don’t feel trepidation about it at all.” (Male, 40-49 years; Focus Group at T3).* Regarding the second theme, several measures and adaptations were implemented on-premises to facilitate adherence with the public health guidance, and these had varied impacts on the participants’ enjoyment of the dining-in experience. Some innovations were viewed favourably by some participants, for example, the implementation of table service and the creative use of outdoor space, e.g., haybales as outdoor seating, although, the enjoyment of eating at outdoor seating was weather dependent. However, some participants found that public health measures such as wearing of masks and the distancing of seating detracted from the enjoyment of dining in, leading some to wait until measures were removed before dining in again.

*The atmosphere, you’re in a social environment and that’s one thing I’ve disliked more so about going to retailers under the restrictions because it doesn’t have the jovial, social atmosphere which is present typically in a retailer or a bar, everyone’s got their masks on, not really looking at each other, not talking. You go out and meet new people, as well as spend time with your friends and that hasn’t been there more recently.” (Male, 50-59 years; Focus Group at T2).* During the second round of focus groups, almost all the participants reported that the enjoyability of the dine-in experience was ‘back to normal’. The participants also enjoyed having the choice of whether to sit in or outdoors.

*“[Facilitator]…how do you feel about eating out at the moment?*

*[Participant] Absolutely fine…Yeah, I I don’t. I I don’t even feel like there’s any restrictions. I mean, you go into some places, there doesn’t seem to be anything sort of in place anymore, which I quite like to be honest with you.”* (Female; 60–69 years; Focus Group at T3).

## 4. Discussion

This study investigated the use of fast food and full-service retailers during various COVID-19 restrictions and changes to takeaway/delivery regulations pertaining to OOH food retailers in England. We collected data across three time points for people living in the North West and North East of England. Our initial sample was 701 people. A total of 615 responded to the follow-up questionnaire at T2 (87.7% of the original sample) and 490 (69.9% of the original sample) responded at T3. In the later waves, fewer women and young people responded, which may have impacted the interpretation of the results in relation to these two characteristics. Most of the participants (54%) reported using new takeaway/delivery services to access food from full-service retailers at least once since their introduction. This was significantly greater than the 19% published in a report by the accounting firm, KPMG [33]. Without access to KPMG’s full survey methods, it was difficult to determine the reasons for this discrepancy. However, their results related to the access to food from retailers and pubs only, whereas ours encompassed all A3/A4 retailers, which also included cafes and bars. Despite this, the use of takeaway/delivery services for full-service retailers at all three survey timepoints was relatively low. Only a small proportion of approx. 25% of the survey participants who ate from full-service retailers used takeaway/delivery services at T1, and a greater use of full-service retailers at T2 and T3 was accompanied with a greater use of dine-in, not takeaway/delivery services. This suggests that the introduction of takeaway/delivery services would be unlikely to have a significant impact on population-level obesity. The reasons for the low uptake of ‘enhanced’ takeaway/delivery services to access full-service retailers emerged in the focus groups. In agreement with a previous study [34], many participants preferred ‘traditional takeaways’ rather than ‘restaurant food’ when having food to be consumed off-premises, and some reported that the poor quality of the delivered food deterred them from using the service again. While some participants were supportive of the new takeaway/delivery services as a temporary ‘bandage solution’ to support full-service retailers while dine-in operations were restricted, when COVID-19 risk was perceived to be low, the participants were eager to dine-in and recapture the social aspect of the eating out experience, despite takeaway/delivery services still being available. One notable exception to this was the view of a focus group member who was shielding and who still valued being able to have food from full-service retailers delivered to their home.

The intake of fast food was consistently more frequent than food from full-service retailers, with approx. 60% of the survey participants consuming fast food at least once a week. This was more frequent than the previous estimates of 21–40% of U.K. adults based on the data collected from 2008–2012 [35,36]. Our findings support the claims that the use of fast food was spurred during the pandemic [37]. It is plausible that this rise in fast food was facilitated by delivery services. A greater use and presence of DFDP delivery drivers was noted by several participants in focus groups, and approx. one-third of all the participants used delivery services to access fast food during all three timepoints. This is consistent with the report findings that in the second quarter of 2021, the DFDP Deliveroo reported a 110% increase in orders across the U.K. and Ireland compared to the first half of 2020 [38]. Some focus group participants reported using these services for the first time during the study period, with some being enticed by introductory promotions—an observation also found in the previous KPMG study [33] and a known strategy used by businesses to grow their customer base [37]. Market intelligence analysts predicted that the rise in delivery services would be a legacy of the pandemic [37]. Our survey data provided some preliminary evidence of this, showing that the intake frequency and use of delivery services to access fast food has been sustained through February 2022, although we cannot determine the proportion of deliveries made by third party DFDPs or in-house services. The observed rise and projected trajectory of the use of delivery services to access fast food occurred even though approx. half of the local government areas in England implemented planning policies that specifically aim to restrict the proliferation of fast food outlets to address obesity [39], e.g., exclusion zones around places for children and families, limiting the maximum number of consecutive takeaway food outlets [39]. The effectiveness of such policies may be undermined by delivery services that facilitate greater access to OOH retailers regardless of physical proximity/access. How delivery services fit within the planning system and the policy challenges they pose requires further research.

Several sociodemographic characteristics were consistently associated with the greater use of retailers across all three timepoints. In line with national survey data [35,40], younger people aged 18–24 years used both fast food and full-service retailers more frequently than older participants. Eating OOH, especially fast food, is linked to social identity and is influenced by subjective social norms among adolescents and young adults [41]. Further, having obesity was consistently associated with a greater intake of food from fast food retailers, but not foods from full-service retailers. While evidence of the relationship between the consumption of OOH foods and weight is equivocal, it does tend to show that BMI is associated positively with the intake of fast food and the association with other retailer types is either weaker [42] or non-significant [43]. It is unlikely that these differences are due to differences in the energy content of menu offerings, which is similar across retailers, if not higher for full-service retailer menu items [44]. An association with fast food but not full-service retailers may be explained by unobserved sociodemographic factors that were not accounted for in our model. For instance, a cross-sectional study in Australian adults found that socioeconomic characteristics were associated with healthy and less healthy takeaway menu choices [45]. While we did not observe a significant difference in the intake frequency between men and women, several female focus group participants mentioned that their domestic and childcare responsibilities prevented them from dedicating time for foodwork and this prompted them to use fast food. The fact that these insights were offered by female participants speaks to the persistent, gendered division of labour, including foodwork [46].

We observed that the use of full-service retailers at T2 and T3 was greater in the participants with higher household incomes and that cost was perceived as a more significant determinant of restaurant use and choice compared to before the pandemic. At the time the focus groups were conducted, inflation rates in the U.K. were rising rapidly [47], and our findings demonstrated the tangible impact this had on people’s decisions surrounding OOH food use. Fast food was consistently used more by those experiencing issues with accessing food for financial reasons. Research suggests that those who are food insecure consume fast food more often as they are more likely to live in high-poverty neighbourhoods with unfettered access to cheap, energy dense foods, such those sold by fast food retailers [48], and constrained access to healthy and nutritious food. There are also suggestions that people experiencing food insecurity use energy dense foods, such as fast foods, as a maladaptive strategy to cope with distress [49].

### 4.1. Strengths and Limitations

This study provided novel insight into consumers’ use of different types of retailers in Northern England during a specific time where the OOH food sector was impacted by various COVID-19 restrictions and changes to takeaway/delivery regulations were implemented. Adopting a mixed-methods approach allowed us to correlate and contextualise the survey findings with qualitative focus group findings. A longitudinal study design allowed us to determine the changes over time as COVID-19 restrictions fluctuated. In terms of limitations, our study sample was recruited via Prolific, which may have introduced some biases, e.g., selection bias—participants who chose to take part in the study may systemically share characteristics and interests. There is also the risk that some participants completed the survey very quickly as a means to make additional money on Prolific. However, we used several attention checks in all the surveys to reduce the risk of using the data provided by these participants. Additionally, although we took measures (stratified sampling) to recruit a diverse sample, our sample was not representative of the population in England. The findings would not be generalisable outside of the study population. However, they are still important for understanding OOH food consumption and access and how it changed with external policy. Additionally, since we were interested in the impact of the new takeaway/delivery services for full-service retailers, we dichotomised the retailers into fast food and full-service retailers, which limited the more granular analysis of other retailer types.

### 4.2. Policy and Practice Considerations, and Future Research

Based on the findings of the current study, we offer the following recommendations for policymakers, practitioners, and researchers in the field of obesity.

1. Delivery services are a growing part of the food environment and are commonly used to access fast food. This needs to be considered when developing local and national planning policies for obesity prevention.
2. Research is needed to understand how delivery services influence the impact of planning policies that restrict the proliferation of fast food outlets.
3. Regarding future crisis planning, if dine-in operations for full-service retailers were restricted in future, reinstating takeaway/delivery services could be considered. They would help preserve business and allow those considered vulnerable to enjoy these retailers.

In the context of the existing literature, our findings also suggest that policies concerning fast food retailers should be prioritised over those concerning full-service retailers, owing to their more frequent consumption and their association with obesity and issues with food access (especially in light of the current ‘cost of living’ crisis in the U.K. [50]). Additionally, since delivery services are currently unregulated and facilitate greater access to outlets regardless of physical proximity/access, upstream interventions that aim to improve the healthiness of menu items must be explored. The feasibility and acceptability of these interventions from business and consumer perspectives need to be evaluated. 

## 5. Conclusions

In the context of fluctuating COVID-19 restrictions and changes to takeaway/delivery regulations, fast food was eaten more frequently than food from full-service retailers in our sample, especially among people with obesity and those with food access issues and was commonly accessed via delivery services. The use of takeaway/delivery services to access food from full-service retailers was low in our sample. Since dining on-premises at full-service retailers is a highly valued part of social life, these services were no longer considered necessary once dine-in restrictions were removed and the risk of COVID-19 was perceived to be low. Policymakers and practitioners need to acknowledge delivery services as a growing part of the food environment and consider this when developing planning policies to address obesity.

## Figures and Tables

**Table 1:** Survey data collected at the three data collection time points (T).

Title 1	T1	T2	T3
Demographics	X		
Fruit and vegetable intake in the preceding 7 days (SFFFQ)	X		
Pre-pandemic frequency of eating from different retailers	X		
Usual alcohol intake (AUDIT-C)	X		
Alcohol intake in the preceding 7 days (modified AUDIT-C)	X	X	X
Physical activity in the preceding 7 days (IPAQ short)	X	X	X
Frequency of eating from fast food and full-service retailers in the preceding 7 days	X	X	X
Use of dine-in, delivery, or takeaway services in the preceding 7 days	X	X	X

Timepoint 1 (T1) from 3–5 May 2021: dine-in services in outdoor spaces only; public health measures enforced on-premises. Timepoint 2 (T2) from 20 May–17 July 2021: indoor spaces reopened; public health measures enforced on-premises. Timepoint 3 (T3) from 11 February–29 March 2022: all legal limits on OOH retailers abandoned. Takeaway/delivery services were available both for fast food and full-service retailers at all time points.

**Table 2:** Characteristics of the survey participants at the three survey timepoints.

	T1 *n* = 701	T2 *n* = 615	T3 *n* = 490
Women (%)	49.9	49.8	47.4
Age (years (SD))	36.0 (14.2)	36.5 (14.3)	38.7 (14.6)
Age groups			
18–24 years (%)	29.2	27.4	21.8
25–49 years (%)	50.8	52.0	52.5
50 years and over (%)	20.0	20.6	25.7
Region			
North East, England	70.3	70.0	73.1
North West, England	29.7	30.0	26.9
White ethnic background (%)	90.6	90.5	92.7
Employment status			
Full-time employed (%)	45.5	44.9	45.3
Part-time employed (%)	19.1	20.2	21.0
Unemployed (%)	10.1	10.3	10.0
Full-time student (%)	16.0	15.8	11.8
Part-time Student (%)	2.9	2.9	2.4
Carer and other (%)	6.4	5.9	9.5
A-level or lower (%)	35.8	35.2	35.3
Issues with access to food			
Definition 1 (%) ^a^	14.7	14.3	13.3
Definition 2 (%) ^b^	8.6	8.3	7.9
Annual household income			
Less than GBP 16,000 (%)	21.4	20.4	21.0
GBP 16,000–29,999 (%)	29.7	30.3	30.2
GBP 30,000–49,999 (%)	26.5	26.8	23.3
GBP 50,000 and over (%)	22.4	22.5	25.5
BMI (kg/m^2^ (SD)) ^c^	26.9 (sd = 6.6)	26.9 (sd = 6.6)	27.1 (sd = 6.6)
Obesity (%)	26.8	26.8	29.0
Meeting five-a-day fruit and vegetable serving guideline (%)	30.2	32.0	31.7
MET hours per week of physical activity ^d^	48.3 (sd = 45.5)	46.5 (sd = 41.3)	48.3 (sd = 45.5)
Able to have food delivered by delivery service e.g., Just Eat (%)	96.0	96.0	95.6

^a^ Answered “often true” or “sometimes true” to any of the items “I/we worried whether my/our food would run out before I/we got money to buy more”; “The food that I/we bought just didn’t last, and I/we didn’t have money to get more”; and “I/we couldn’t afford to eat balanced meals”. ^b^ Answered “often true” or “sometimes true” to any two of the items “I/we worried whether my/our food would run out before I/we got money to buy more”; “The food that I/we bought just didn’t last, and I/we didn’t have money to get more”; and “I/we couldn’t afford to eat balanced meals.” ^c^ *n*= 698 at Survey 1. ^d^ Physical activity included the time spent walking, and in moderate and vigorous physical activity.

**Table 3:** Characteristics of the focus group participants at T2 and T3.

	T2 *n* = 22	T3 *n* = 12
Women (*n*; %)	12; 55	6; 50
Age group (n; %)		
<30 years	9; 41	3; 25
30–39 years	2; 9	0; 0
40–49 years	3; 14	3; 25
50–59 years	4; 18	4; 33
≥60 years	4; 18	2; 17
Annual household income (n; %)		
<GBP 10,000	2; 9	0; 0
GBP 10,000–19,999	4; 18	2; 17
GBP 20,000–29,999	4; 18	1; 8
GBP 30,000–39,999	3; 14	1; 8
GBP 40,000–49,999	2; 9	3; 25
GBP 50,000–99,999	5; 23	5; 42
≥GBP 100,000	2; 9	0; 0

**Table 4:** Ordered logit model of the determinants of eating from fast food retailers once or more a week.

	T1	T2	T3
18–24 years old	Reference	Reference	Reference
25–49 years old	0.486 ***	0.478 ***	0.522 **
	**(0.111)**	**(0.121)**	**(0.158)**
50 and older	0.177 ***	0.164 ***	0.225 ***
	**(0.0510)**	**(0.0509)**	**(0.0791)**
North East	Reference	Reference	Reference
North West	0.767	1.211	1.386
	(0.154)	(0.259)	(0.334)
Degree or higher	Reference	Reference	Reference
A-level or lower	1.040	1.390 *	0.946
	(0.172)	(0.246)	(0.184)
Healthy BMI	Reference	Reference	Reference
Overweight BMI	1.193	1.186	1.176
	(0.229)	(0.239)	(0.271)
Obese BMI	1.607 **	2.212 ***	1.846 ***
	**(0.310)**	**(0.460)**	**(0.420)**
Household income < GBP 16K	Reference	Reference	Reference
GBP 16–29K	1.044	1.431	1.188
	(0.255)	(0.376)	(0.345)
GBP 30–49K	1.383	1.521	1.317
	(0.352)	(0.418)	(0.396)
GBP 50K or greater	1.651*	1.558	1.097
	(0.444)	(0.453)	(0.353)
No food access issues	Reference	Reference	Reference
Food access	1.652 **	2.597 ***	2.107 **
	**(0.400)**	**(0.661)**	**(0.619)**
Not in employment/Other	Reference	Reference	Reference
Full-time employed	1.516 *	1.385	1.605
	(0.369)	(0.353)	(0.466)
Part-time employed	1.242	0.990	1.224
	(0.312)	(0.257)	(0.352)
Student	1.199	0.881	1.243
	(0.324)	(0.251)	(0.471)
Carer	3.510 ***	1.094	1.936
	**(1.318)**	(0.422)	(0.856)
Male	Reference	Reference	Reference
Female	0.967	1.153	0.896
	(0.163)	(0.208)	(0.180)
/cut1	0.469	0.683	0.784
	(0.259)	(0.441)	(0.543)
/cut2	3.076 **	4.123 **	4.004 **
	(1.701)	(2.670)	(2.789)
/cut3	12.02 ***	13.93 ***	12.60 ***
	(6.812)	(9.132)	(8.921)
Observations	599	550	427

Results presented as odds ratios (standard error). The figures in bold type indicate a statistical significance at *p* < 0.05; * *p* < 0.10, ** *p* < 0.05, *** *p* < 0.01. Timepoint 1 (T1) from 3–5 May 2021: dine-in services in outdoor spaces only; public health measures enforced on-premises. Timepoint 2 (T2) from 20 May–17 July 2021: indoor spaces reopened; public health measures enforced on-premises. Timepoint 3 (T3) from 11 February–29 March 2022: all legal limits on OOH retailers abandoned. Takeaway/delivery services were available both for fast food and full-service retailers at all time points.

**Table 5:** Ordered logit model of the determinants of eating from full-service retailers once or more a week.

	T1	T2	T3
18–24 years old	Reference	Reference	Reference
25–49 years old	0.574 **	0.449 ***	0.553 *
	**(0.149)**	**(0.119)**	**(0.175)**
50 and older	0.336 ***	0.375 ***	0.399 ***
	**(0.116)**	**(0.118)**	**(0.141)**
North East	Reference	Reference	Reference
North West	0.683	1.289	0.914
	(0.167)	(0.293)	(0.233)
Degree or higher	Reference	Reference	Reference
A-level or lower	0.636 **	0.951	0.818
	**(0.133)**	(0.175)	(0.167)
Healthy BMI	Reference	Reference	Reference
Overweight BMI	1.070	0.913	1.140
	(0.243)	(0.190)	(0.268)
Obese BMI	0.840	0.905	1.271
	(0.203)	(0.195)	(0.298)
Household income < GBP 16K	Reference	Reference	Reference
GBP 16–29K	1.334	1.637 *	1.036
	(0.402)	(0.445)	(0.313)
GBP 30–49K	1.305	1.796 **	1.288
	(0.406)	**(0.524)**	(0.402)
GBP 50K or greater	1.320	2.406 ***	1.925 *
	(0.427)	**(0.731)**	(0.646)
No food access issues	Reference	Reference	Reference
Food access	1.077	1.609 *	1.245
	(0.318)	(0.433)	(0.384)
Not in employment/Other	Reference	Reference	Reference
Full-time employed	1.175	1.329	1.340
	(0.344)	(0.348)	(0.392)
Part-time employed	1.024	1.546	1.035
	(0.312)	(0.416)	(0.307)
Student	1.095	1.499	1.498
	(0.346)	(0.454)	(0.571)
Carer	0.207 **	0.849	1.035
	**(0.158)**	(0.361)	(0.436)
Male	Reference	Reference	Reference
Female	1.241	1.300	1.130
	(0.247)	(0.248)	(0.234)
/cut1	1.610	2.303	1.059
	(1.032)	(1.480)	(0.759)
/cut2	7.042 ***	16.41 ***	5.653 **
	(4.583)	(10.75)	(4.082)
/cut3	22.68 ***	53.15 ***	21.48 ***
	(15.49)	(35.92)	(16.04)
Observations	599	524	427

Results presented as odds ratios (standard error). The figures in bold type indicate a statistical significance at *p* < 0.05; * *p* < 0.10, ** *p* < 0.05, *** *p* < 0.01. Timepoint 1 (T1) from 3–5 May 2021: dine-in services in outdoor spaces only; public health measures enforced on-premises. Timepoint 2 (T2) from 20 May–17 July 2021: indoor spaces reopened; public health measures enforced on-premises. Timepoint 3 (T3) from 11 February–29 March 2022: all legal limits on OOH retailers abandoned. Takeaway/delivery services were available both for fast food and full-service retailers at all time points.

## Data Availability

The datasets used and/or analysed during the current study are available from the corresponding authors upon reasonable request.

## Supplementary Materials

The following supporting information can be downloaded at: https://www.mdpi.com/article/10.3390/nu15163636/s1, File S1: Baseline survey; File S2: Focus group topic guide.
