# Supplementary material for: The Impact of COVID-19 Restrictions and Changes to Takeaway Regulations in England on Consumers’ Intake and Methods of Accessing Out-of-Home Foods: A Longitudinal, Mixed-Methods Study
Source: Nutrients. 2023 Aug 18;15(16):3636. doi: 10.3390/nu15163636 (PMC10459227; doi:10.3390/nu15163636)
Supplement: Supplementary file 1 [file nutrients-15-03636-s001.zip › Additional File S1.pdf]

Additional File S1. Survey at Timepoint 1.

Informed Consent

.
Thank you for giving your time and effort to help with our study, we really appreciate it.

This is **Survey 1** of two surveys in our study. It will take around **15 minutes** and reimbursement is **£2.25**. We would like to ask you questions about: your background e.g. your employment, your height and weight and your education; your general eating, drinking and exercise patterns; eating food from specific types of food outlets. The final question in Survey 1 will ask if you are interested in taking part in a related focus group over the next few weeks.

After you complete Survey 1, you will be invited to take part in **Survey 2** around 6 weeks later. It will take about **10 minutes** and reimbursement for Survey 2 is **£1.55**.

You can stop the surveys at any time. If you stop and do not complete the survey, that is fine, please just remember to return your submission on Prolific by selecting the 'Stop without completing' button.

Thank you again for giving your time and important insights.

This project is funded by Public Health England and is being delivered by Newcastle University in collaboration with Teesside University. The team at Newcastle University is made up of Drs Mackenzie Fong, Steph Scott and Shelina Visram. This project has been approved by Newcastle University ethics (REF 10457/2020). For more information contact the study lead, Dr Mackenzie Fong: mackenzie.fong@newcastle.ac.uk

- ◯ **I consent** to begin the study
- ◯ **I do not consent** to begin the study

Does not consent

. As you do not wish to participate in this study, please return your submission on Prolific by selecting the 'Stop without completing' button.

Screener validation

. Firstly, we would like to find out a bit about you.

Q1. Enter your Prolific ID here:

${e://Field/PROLIFIC_PID}

Q2.
Please select your age group.

- ◯ 18-24 years
- ◯ 25-49 years
- ◯ 50 years and over

Q3. What sex were you assigned at birth, such as on an original birth certificate?

- ◯ Male
- ◯ Female
- ◯ Prefer not to say

Q4. What UK area do you currently live in?

- ◯ North East, England (Tees Valley, Durham, Northumberland and Tyne and Wear)

- ◯ North West, England (Cumbria, Greater Manchester, Lancashire, Merseyside)
- ◯ Yorkshire and the Humber, England (East Riding, North Lincolnshire and Yorkshire)
- ◯ East Midlands, England (Derbyshire and Nottinghamshire, Leicestershire, Rutland and Northamptonshire, Lincolnshire)
- ◯ West Midlands, England (Herefordshire, Worcestershire and Warwickshire, Shropshire and Staffordshire, West Midlands)
- ◯ East of England (East Anglia, Bedfordshire and Hertfordshire, Essex)
- ◯ London, England
- ◯ South East, England (Berkshire, Buckinghamshire, and Oxfordshire, Surrey, Sussex, Kent, Hampshire and Isle of Wight)
- ◯ South West, England (Gloucestershire, Wiltshire and Bristol/Bath area, Dorset and Somerset, Cornwall and Isles of Scilly, Devon)
- ◯ Wales
- ◯ Scotland
- ◯ Northern Island

Q5. What is your total household income per year, including all earners in your household (after tax) in GBP? If you need to convert from another currency you can find a converter here.

- ◯ Less than £10,000
- ◯ £10,000 - £15,999
- ◯ £16,000 - £19,999
- ◯ £20,000 - £29,999
- ◯ £30,000 - £39,999
- ◯ £40,000 - £49,999
- ◯ £50,000 - £59,999
- ◯ £60,000 - £69,999
- ◯ £70,000 - £79,999
- ◯ £80,000 - £89,999
- ◯ £90,000 - £99,999
- ◯ £100,000 - £149,999
- ◯ More than £150,000
- ◯ Rather not say

Inconsistent screening responses

Q9. You are ineligible for this study, as you have provided information which is inconsistent with your Prolific prescreening responses. Please return your submission on Prolific by selecting the 'Stop without completing' button.

Questions

Q6. What is your ethnicity?

- ◯ White
- ◯ Mixed or Multiple ethnic groups
- ◯ Asian or Asian British
- ◯ Black, African, Caribbean or Black British
- ◯ Other ethnic group

Q7.
What is your highest educational qualification? If you are a student please select the qualification being studied for.

- ◯ No formal qualifications
- ◯ 1-3 GCSEs or equivalent
- ◯ 4+ GSCEs or equivalent
- ◯ A level or equivalent
- ◯ Certificate of higher education (CertHE) or equivalent
- ◯ Diploma of higher education (DipHE) or equivalent
- ◯ Bachelor or equivalent
- ◯ Master’s degree or equivalent
- ◯ Doctorate or equivalent

Q8.
Which of these describes your **current** employment situation?

Select all that apply.

- ☐ Full-time employed
- ☐ Part-time employed
- ☐ Not employed for pay
- ☐ Caregiver (e.g., children, elderly)
- ☐ Full-time student
- ☐ Part-time student
- ☐ Other

Q9. What is the first part of your postcode? For example, if your full postcode is AB12 3CD, just write AB12.

- ◯ Postcode
- ◯ Rather not say

Q10. The next question will ask for your height. How would you prefer to answer?

- ◯ In feet and inches
- ◯ In centimetres

Q10a. How tall in feet and inches are you **without** shoes on?

	Feet	Inches
Height		

Q10a. How tall in centimetres are you **without** shoes on?

Please answer to the nearest centimetre.

Q11. The next question will ask for your weight. How would you prefer to answer?

- ◯ In stones and pounds

○ In kilograms

Q11a. How much do you weigh in stones and pounds **without clothes or shoes**?

	Stones	Pounds
Weight		

Q11a. How much do you weigh in kilograms **without clothes or shoes**?

Please estimate your answer to the nearest kilogram.

Q12. Next, you will see some statements that people have made about their food situation.

Please let us know whether each statement was often true, sometimes true, or never true for you/your household since the **start of the pandemic** (since March 2020).

	Often true	Sometimes true	Never true	Don't know	Prefer not to say
I/we worried whether my/our food would run out before I/we got money to buy more.	○	○	○	○	○
The food that I/we bought just didn't last, and I/we didn't have money to get more.	○	○	○	○	○
I/we couldn't afford to eat balanced meals.	○	○	○	○	○

Q13. For what reasons have you found it difficult to access food during lockdown (since March 2020)?

Select all that apply.

☐ I/We did not have enough money for food

☐ The shops did not have the food I/we needed

☐ I/We could not go out and did not have any other way to get the food we needed

☐ I/We **have not** experienced issues with accessing food during lockdown

☐ For other reasons (please provide more information)

☐ Don't know

☐ Prefer not to say

Q14. Are secondary children in your household eligible for Free School Meals?

○ Yes

○ No

○ Don't know

○ Prefer not to say

○ There are no secondary school children in my household

Q15. The following questions ask about some foods & drinks you might have during a **'typical' week**, over the past month or so. Do not be concerned if some things you eat or drink are not mentioned. Please select **how often** you eat **at least one portion** of the

following.

Tips:

- Examples of one portion of fruit/fruit juice are: a handful of grapes, an orange, a glass of fruit juice, a handful of dried fruits.
- Examples of one portion of salad and vegetables are: 3 heaped tablespoons of carrots, a side salad, 2 spears of broccoli

	Rarely or never	Less than 1 a week	Once a week	2-3 times a week	4-6 times a week	1-2 times a day	3-4 times a day	5+ a day	Don't know
Fruit (tinned/fresh)	○	○	○	○	○	○	○	○	○
Fruit juice (not cordial or squash)	○	○	○	○	○	○	○	○	○
Salad (not garnish added to sandwiches)	○	○	○	○	○	○	○	○	○
Vegetables (tinned/frozen but not potatoes)	○	○	○	○	○	○	○	○	○
It's important that you pay attention to this study. Please select 'Once a week'	○	○	○	○	○	○	○	○	○

Q16. On average, **how many** portions of fruit do you eat a day?

Tip:

- Examples of one portion include: a handful of grapes, an orange, a glass of fruit juice, a handful of dried fruits

0	1	2	3	4	5 or more	Don't know
○	○	○	○	○	○	○

Q17. On average, **how many** portions of vegetables do you eat a day?

Tip:

- Examples of one portion include: 3 heaped tablespoons of carrots, a side salad, 2 spears of broccoli

0	1	2	3	4	5 or more	Don't know
○	○	○	○	○	○	○

. These next few questions will ask about your **typical pattern** of drinking alcohol.

Q18.
How often do you have a drink containing alcohol?

- ○ Never
- ○ Monthly or less
- ○ 2-4 times per month
- ○ 2 to 3 times per week
- ○ 4 or more times per week

Q18a. How many units of alcohol do you drink on a **typical day** when you are drinking? The image below can help you estimate.

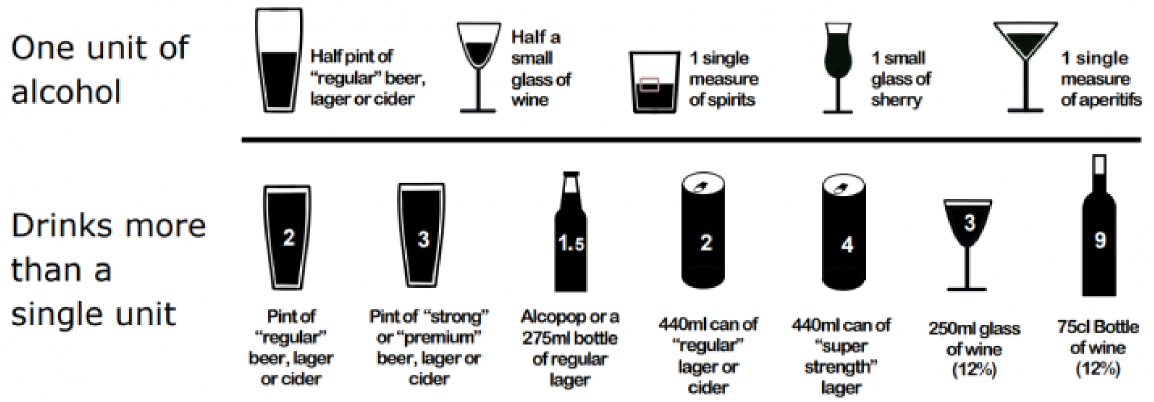

- ◯ 0 to 2 units
- ◯ 3 to 4 units
- ◯ 5 to 6 units
- ◯ 7 to 9 units
- ◯ 10 or more units

Q18b. How often have you had **6 or more** units on a single occasion in the last year?

- ◯ Never
- ◯ Less than monthly
- ◯ Monthly
- ◯ Weekly
- ◯ Daily or almost daily

Q18b. How often have you had **8 or more** units on a single occasion in the last year?

- ◯ Never
- ◯ Less than monthly
- ◯ Monthly
- ◯ Weekly
- ◯ Daily or almost daily

. These next few questions will now ask about drinking alcohol over the **past 7 days**.

Q19. How many days did you have a drink containing alcohol in the **past 7 days**?

None	1 day	2 days	3 days	4 days	5 days	6 days	7 days
◯	◯	◯	◯	◯	◯	◯	◯

Q19a. How many units of alcohol **in total** did you have in the **past 7 days**? The image below can help you estimate.

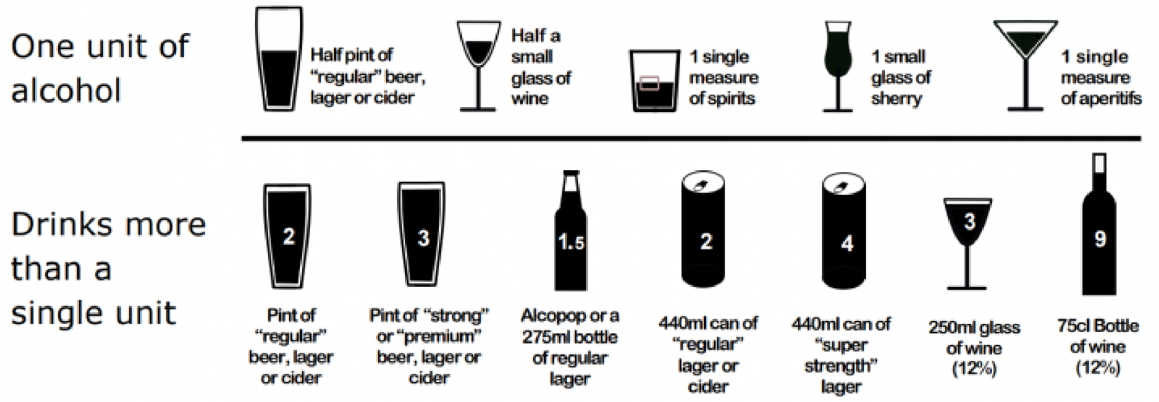

- ◯ 1 to 7 units
- ◯ 8 to 14 units
- ◯ 15 to 21 units
- ◯ More than 21 units

Q19b. How often did you have **6 or more** units on a single occasion in the **last 7 days**?

0 times	1 times	2 times	3 times	4 times	5 times or more
◯	◯	◯	◯	◯	◯

Q19b. How often did you have **8 or more units** on a single occasion in the **last 7 days**?

0 times	1 time	2 times	3 times	4 times	5 times or more
◯	◯	◯	◯	◯	◯

Q19c.
When asked for your favourite animal you must enter the word 'unicorn' in the text box below.

Based on the text you read above, what animal have you been asked to enter?

. Next, we would like to find out about eating **hot foods** prepared outside the home from these 2 groups of food outlets**:**

1. Fast food outlets

- Hot foods are usually **eaten off-premises** due to **limited seating**
- Hot foods are usually **purchased at the till**
- Examples include chains like Greggs, KFC, Dominos, and also independent outlets like your local fish 'n chip shop
  Foods are generally pre-prepared and require minimal assembly. Examples of common hot foods sold at fast food
- outlets are: burgers, fried chicken, fish 'n chips, pizza, kebabs, burritos, cooked sausage rolls/pies, curries and rice/noodle dishes.
  Here are some photos of typical fast food outlets:
-

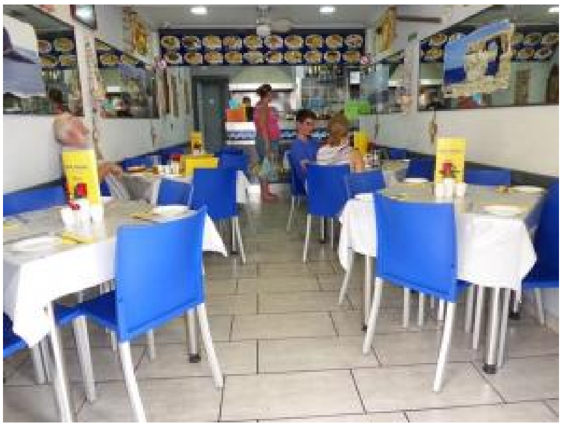

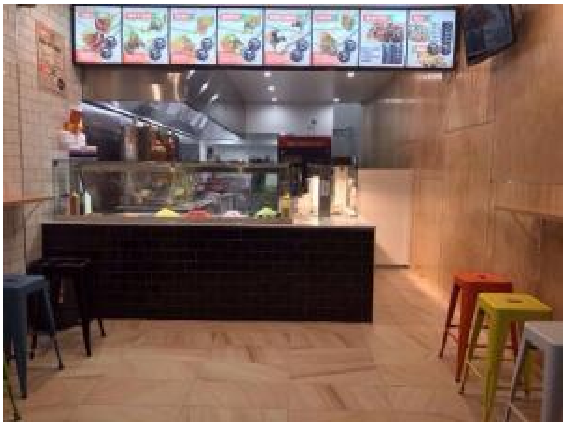

2. Restaurants, pubs, bars & cafes

- Hot foods are eaten on-premises as there is **plenty of seating**
- Foods require more preparation, cooking and assembly, and are generally 'made to order'
- These outlets have been able to offer takeaway and delivery services during the pandemic
- Here are some photos of typical outlets in this group:

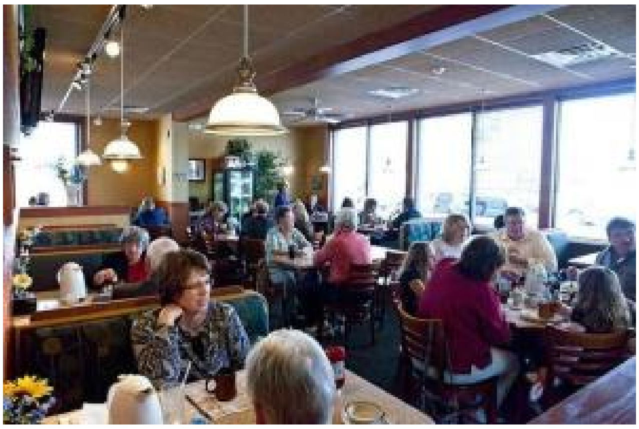

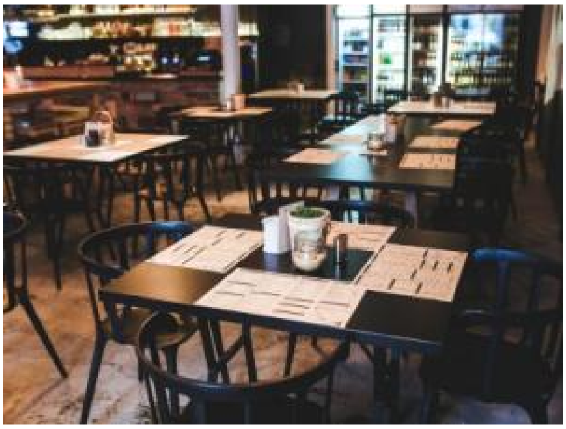

These notes will be displayed underneath the next few questions to help you answer.

Q20.
How often did you eat hot foods from these outlets **before the pandemic** (before March 2020)?

Tips:

- Only answer for **hot foods** that can be **eaten immediately** after purchase (you don't even need to heat them up or recook them)
- Think about **all eating occasions** throughout the day (breakfast, lunch, evening meal and in between)
- If you need reminding about these outlets, there is some helpful text at the bottom of the page

	Less than once a month	1-2 times a month	3-4 times a month	1-2 times a week	3-4 times a week	5-6 times a week	7 or more times a week
Fast food outlets	○	○	○	○	○	○	○
Restaurants, bars, pubs & cafes	○	○	○	○	○	○	○

.
1. Fast food outlets

- Hot foods are usually **eaten off-premises** due to **limited seating**
- Hot foods are usually **purchased at the till**
- Examples include chains like Nandos, Greggs, KFC, Dominos, and also independent outlets like your local fish 'n chip shop
- Foods are generally **pre-prepared** and require minimal assembly. Examples of common hot foods sold at fast food outlets are: burgers, fried chicken, fish 'n chips, pizza, kebabs, burritos, cooked sausage rolls/pies, curries and rice/noodle dishes.

2. Restaurants, pubs, bars & cafes

- Hot foods are eaten on-premises as there is **plenty of seating**

- Foods require more preparation, cooking and assembly, and are generally 'made to order'
- These outlets have been able to offer takeaway and delivery services during the pandemic

Q21.
How often did you eat hot foods from these outlets over the **past 7 days**?

Tips:

- Only answer for **hot foods** that can be **eaten immediately** after purchase (you don't even need to heat them up or recook them)
- Think about **all eating occasions** throughout the day (breakfast, lunch, evening meal and in between)
- If you need reminding about these outlets, there is some helpful text at the bottom of the page

	0 times	1 time	2 times	3 times	4 times	5 times	6 times	7 or more times
Fast food outlets	○	○	○	○	○	○	○	○
Restaurants, bars, pubs & cafes	○	○	○	○	○	○	○	○

.
1. Fast food outlets

- Hot foods are usually **eaten off-premises** due to **limited seating**
- Hot foods are usually **purchased at the till**
- Examples include chains like Nandos, Greggs, KFC, Dominos, and also independent outlets like your local fish 'n chip shop
- Foods are generally **pre-prepared** and require minimal assembly. Examples of common hot foods sold at fast food outlets are: burgers, fried chicken, fish 'n chips, pizza, kebabs, burritos, cooked sausage rolls/pies, curries and rice/noodle dishes.

2. Restaurants, pubs, bars & cafes

- Hot foods are eaten on-premises as there is **plenty of seating**
- Foods require more preparation, cooking and assembly, and are generally 'made to order'
- These outlets have been able to offer takeaway and delivery services during the pandemic

Q21a.
How did you access **hot foods** from **fast food outlets** over the **past 7 days**?

Select all that apply.

- ☐ Delivery
- ☐ Collected from the outlet (takeaway/carryout)
- ☐ Dined in
- ☐ Don't know

Q21b.
How did you access **hot foods** from **restaurants, bars, pubs & cafes** over the **past 7 days**?

Select all that apply.

- ☐ Delivered to me
- ☐ Collected from the outlet (takeaway/carryout)
- ☐ Dined in
- ☐ Don't know

Q21c. Are you able to have food delivered to your house by a food delivery service e.g. Deliveroo, Uber Eats, Just Eat?

- ○ Yes
- ○ No
- ○ Don't know

Q21d. Think about the **most recent time** you ate hot food from a **fast food outlet.** Please provide a brief description of the **type and amount** of food and drink you had on this occasion in the corresponding text boxes.

Tip:

- Include **all** foods and drinks you had on this occasion, not just those you purchased from the outlet e.g. if you got delivery/takeaway and you drank Coke that you had at home, you would also include this.
- An example is show below:

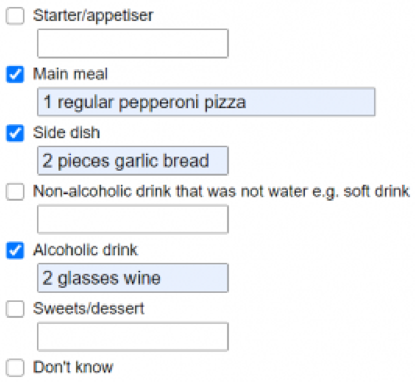

☐ Starter/appetiser

☐ Main meal

☐ Side dish

☐ Non-alcoholic drink that was not water e.g. soft drink

☐ Alcoholic drink

☐ Sweets/dessert

☐ Don't know

Q21e. Think about the **most recent time** you ate hot food from a **restaurant, bar, pub or cafe**. Please provide a brief description of the **type and amount** of food and drink you had on this occasion in the corresponding text boxes.

Tip:

- Include **all** foods and drinks you had on this occasion, not just those you purchased from the outlet e.g. if you got delivery/takeaway and you drank Coke that you had at home, you would also include this.
- An example is show below:

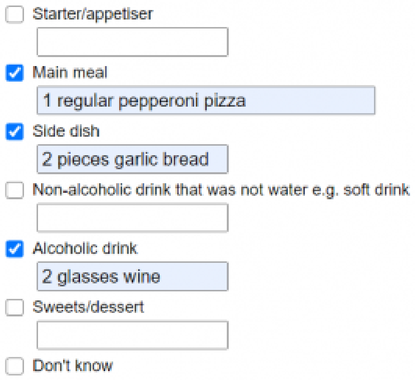

☐ Starter/appetiser

☐ Main meal

- ☐ Side dish
- ☐ Non-alcoholic drink that was not water e.g. soft drink
- ☐ Alcoholic drink
- ☐ Sweets/dessert
- ☐ Don't know

. Next, we would like to find out about the kinds of **physical activities** you do as part of your everyday life. The next few questions will ask you about the time you spent being physically active in the last 7 days.

Please answer each question even if you do not consider yourself to be an active person.

Please think about the activities you do at work, as part of your house and yard work, to get from place to place, and in your spare time for recreation, exercise or sport.

.
Think about all the **vigorous activities** that you did in the last 7 days. Vigorous physical activities refer to activities that take hard physical effort and make you **breathe much harder than normal**.

Q22.
During the **last 7 days**, on how many days did you do **vigorous physical activities** like heavy lifting, digging, aerobics, or fast bicycling?

Think only about those physical activities that you did for **at least 10 minutes at a time**.

None	1 day	2 days	3 days	4 days	5 days	6 days	7 days
○	○	○	○	○	○	○	○

Q22a. How much time did you usually spend doing vigorous physical activities on one of those days?

Please give your answer in **hours and minutes** e.g. 1 hour and 15 minutes.

	Hours per day	Minutes per day
Time spent doing vigorous activities	⌄	⌄

.
Now, think about all the **moderate activities** that you did in the last 7 days. Moderate activities refer to activities that take moderate physical effort and make you **breathe somewhat harder than normal**.

Q23.
During the **last 7 days**, on how many days did you do **moderate physical activities** like carrying light loads, bicycling at a regular pace, or doubles tennis? **Do not include walking**.

Think only about those physical activities that you did for **at least 10 minutes at a time**.

None	1 day	2 days	3 days	4 days	5 days	6 days	7 days
○	○	○	○	○	○	○	○

Q23a. How much time did you usually spend doing moderate physical activities on one of those days?

Please give your answer in **hours and minutes** e.g. 1 hour and 15 minutes.

	Hours per day	Minutes per day
Time spent doing moderate activites		

.
Now, think about the time you spent **walking in the last 7 days**. This includes at work and at home, walking to travel from place to place, and any other walking that you have done solely for recreation, sport, exercise, or leisure.

Q24.
During the last 7 days, on how many days did you walk for **at least 10 minutes at a time**?

None	1 day	2 days	3 days	4 days	5 days	6 days	7 days
◯	◯	◯	◯	◯	◯	◯	◯

Q24a. How much time did you usually spend walking on one of those days?

Please give your answer in **hours and minutes** e.g. 1 hour and 15 minutes.

	Hours per day	Minutes per day
Time spent walking		

.
This next question is about the time you spent **sitting on weekdays** during the last 7 days. Include time spent at work, at home, while doing course work and during leisure time. This may include time spent sitting at a desk, visiting friends, reading, or sitting or lying down to watch television.

Q25. During the last 7 days, how much time did you spend sitting on a week day?

Please give your answer in **hours and minutes** e.g. 1 hour and 15 minutes.

	Hours per day	Minutes per day
Time spent sitting		

Q26.

Thank you!

Thank you very much for giving your time and effort to answer our questions. We really appreciate it!

Lastly, we would like to know if you would be interested in taking part in a **focus group** that is related to this survey. You would meet virtually with around 3-5 other participants and 1-2 researchers sometime over the next few weeks. The discussion would last around one hour and you would be reimbursed for your time (**£15/hour**).

- ◯ I **am interested** in taking part in a focus group (if there are still spots available you will be provided with access to another survey containing further information)
- ◯ I am **not interested** in taking part in a focus group
