# Supplementary material for: The Impact of COVID-19 Restrictions and Changes to Takeaway Regulations in England on Consumers’ Intake and Methods of Accessing Out-of-Home Foods: A Longitudinal, Mixed-Methods Study
Source: Nutrients. 2023 Aug 18;15(16):3636. doi: 10.3390/nu15163636 (PMC10459227; doi:10.3390/nu15163636)
Supplement: Supplementary file 1 [file nutrients-15-03636-s001.zip › Additional File S2.pdf]

Additional File S2. Focus group topic guide at Timepoint 1.

Note: As per standard procedure, this focus group schedule is developmental in accordance with the iterative conduct and analysis of the interviews. Questions will be tailored to the specific answers of each interviewee.

Focus group setting

- Virtual (phone or video call)

Focus group format

- Introduction to the study (~5-10 minutes)
- Focus Group (~50 minutes)

Introduction (5 minutes)

1. Thank you for taking the time to speak with us today.
2. Introduce self/role
3. We'd like to find out more about your experiences of eating foods that have been prepared outside of your home. This includes hot foods that are ready to eat, prepared in outlets such as fast-food outlets, restaurants, bars, pubs, cafes etc.
4. We are interested in your experiences before and during the pandemic, up to the present.
5. Explain confidentiality (guaranteed except where there is a perceived risk of harm to self or others)
6. There are no right or wrong answers, take your time and if you would like a break, just say
7. If you'd like to say something, please use the raise hand function – or if you don't have the function, just put a message in the chat
8. You don't have to answer every question. If there is nothing you'd like to say, that's fine. If you would prefer to say something in the chat that's fine.
9. Being able to see faces can make the conversation a bit more natural etc. But you don't have to if you don't want to.
10. The focus group is audio recorded but all details will be kept confidential and transcripts will be anonymised
11. Confirm that everyone has read the study description on Prolific.
12. You have provided your consent online. If you haven't provided your consent yet, or you've changed your mind, please just let us know. You can do so in the Chat.

Introduction and icebreaker session (10 minutes)

- First name (if you want)
- Where you're from
- Food question
    - if you could eat only one food for the rest of your life what would it be?
    - What is the weirdest food combination you eat?

Experiences of 'eating out' (pre-pandemic) (10-15 minutes)

1. Can you tell us a little bit about how you'd define 'eating out', what would you include?
2. Can you tell us a little bit about your experiences of 'eating out' including takeaway and delivery, before the pandemic?
    - Can probe on: frequency, type of establishment/type of food/meal of the day, perceived healthiness, reasons/occasions
3. What do you think tends to influence your decisions about where to eat?
    - Can probe on: emotions, routine, social/cultural, opportunity / necessity/practical i.e. near to work/no time to cook, price, location
4. What do you miss the most about 'eating out' before the pandemic? Why?

Experiences of 'eating out' (during the pandemic to the present) (25-30 minutes)

1. Can you tell us a little bit about your experiences of 'eating out' including getting takeaway and delivery during the pandemic?
    - Can probe on: how food is accessed e.g. Deliveroo, collection, dine-in
2. Since the pandemic, food outlets like restaurants, bars, pubs and cafes have been able to offer takeaway and delivery. Has being able to get takeaway or delivery from these outlets changed your eating behaviour compared to before the pandemic?
    - Can probe on: frequency, how food is accessed e.g. Deliveroo, type of establishment/type of food/meal of the day
    - Can probe on: do you think these changes will last? Why/why not?
3. What do you think influenced this?
    - Can probe on: location, greater choice/access, supporting local businesses, having children
4. Were your experiences / behaviours the same throughout or, for example, was Lockdown 1 different to Lockdown 2? Why?
5. Can you tell us about your responses to easing of restrictions in the UK, first in Summer 2020 and now across April and May 2021? Has this changed your behaviour/experiences?
    - Can probe on: Eat Out to Help Out

6. Have you eaten out at an establishment in the past few weeks since restrictions have eased? What was this experience like?
   - Can probe on: accessibility (i.e. trying to make a reservation), enjoyability/atmosphere, ordering systems, COVID-safety measures
7. Does your eating behaviour differ depending on whether you eat out at an establishment, or if you have takeaway or delivery at home?
   - can probe on: amount ordered/eaten (e.g. ordering starters, desserts) drinking alcohol, external influences e.g. social, environmental (smell and sight of food), consideration of healthfulness/calories

Changes to behaviours (10-15 minutes)

8. Do you feel that there have been any changes – large or small – in how you eat during this time?
   - Can probe on: volume of food, type of food, nutrition/health impacts, time of day
9. Have you notice any impacts upon your health?
   - Can probe on: mental health, weight, exercise, sleep
10. Can you tell us a little bit about your alcohol consumption over the last 12-14 months?
    - Can probe on: changes, links to food consumption
11. Do you think your relationship with food has changed?

Closing questions (5 minutes)

1. These are all the questions we had for you today. Is there anything else you would like to discuss that we haven't covered already? Do you have any questions for us?
2. Thank you again for your time
3. Get Prolific IDs
